# Abrine, an IDO1 inhibitor, suppresses the immune escape and enhances the immunotherapy of anti-PD-1 antibody in hepatocellular carcinoma

**DOI:** 10.3389/fimmu.2023.1185985

**Published:** 2023-05-24

**Authors:** Xiaowei Liang, Hongwei Gao, Jian Xiao, Shan Han, Jia He, Renyikun Yuan, Shilin Yang, Chun Yao

**Affiliations:** ^1^College of Pharmacy, Guangxi University of Chinese Medicine, Nanning, China; ^2^Engineering Research Center in Ministry of Education for Innovative Drugs of Traditional Chinese Medicine and Zhuang Yao Medicine, Nanning, China

**Keywords:** IDO1, Abrine, CD47, PD-L1, m^6^A RNA modification, immune escape, hepatocellular carcinoma

## Abstract

**Background:**

Indoleamine-2,3-dioxygenase 1 (IDO1) is responsible for tumor immune escape by regulating T cell-associated immune responses and promoting the activation of immunosuppressive. Given the vital role of IDO1 in immune response, further investigation on the regulation of IDO1 in tumors is needed.

**Methods:**

Herein, we used ELISA kit to detect the interferon-gamma (IFN-γ), Tryptophan (Trp), and kynurenic acid (Kyn) levels; western blot, Flow cytometry, and immunofluorescence assays detected the expression of the proteins; Molecular docking assay, SPR assay and Cellular Thermal Shift Assay (CETSA) were used to detect the interaction between IDO1 and Abrine; nano live label-free system was used to detect the phagocytosis activity; tumor xenografts animal experiments were used to explore the anti-tumor effect of Abrine; flow cytometry detected the immune cells changes.

**Results:**

The important immune and inflammatory response cytokine interferon-gamma (IFN-γ) up-regulated the IDO1 expression in cancer cells through the methylation of 6-methyladenosine (m6A) m6A modification of RNA, metabolism of Trp into Kyn, and JAK1/STAT1 signaling pathway, which could be inhibited by IDO1 inhibitor Abrine. CD47 is IFN-γ-stimulated genes (ISGs) and prevents the phagocytosis of macrophages, leading to the cancer immune escape, and this effect could be inhibited by Abrine both in vivo and in vitro. The PD-1/PD-L1 axis is an important immune checkpoint in regulating immune response, overexpression of PD-1 or PD-L1 promotes immune suppression, while in this study Abrine could inhibit the expression of PD-L1 in cancer cells or tumor tissue. The combination treatment of Abrine and anti-PD-1 antibody has a synergistic effect on suppressing the tumor growth through up-regulating CD4^+^ or CD8^+^ T cells, down-regulating the Foxp3^+^ Treg cells, and inhibiting the expression of IDO1, CD47, and PD-L1.

**Conclusion:**

Overall, this study reveals that Abrine as an IDO1 inhibitor has an inhibition effect on immune escape and has a synergistic effect with the anti-PD-1 antibody on the treatment of HCC.

## 1 Introduction

Epigenetics is the stable inheritance that changes gene expression or function by regulating the interaction between the genome and the environment without changing the basic sequence of DNA, mainly including DNA methylation, histone modification, chromatin reorganization, and RNA modification (1). The methylation of 6-methyladenosine (m^6^A) is the most abundant epitranscriptomic modification in eukaryotic mRNA, which plays an important role in affecting oncogenic and tumor suppressor networks and regulating tumor immunogenicity (2).

Primary liver cancer is a high-incidence and malignant tumor in the world and the second leading cause of cancer deaths worldwide (3). Hepatocellular carcinoma (HCC) is the most common type of primary liver cancer and most commonly occurs in patients with chronic liver disease, such as cirrhosis caused by hepatitis B or C infection (4). The updates of HCC treatment methods mainly include surgical resection, liver transplantation, local ablative therapy, systemic therapy, etc (5). Indoleamine-2,3-dioxygenase 1 (IDO1) is an intracellular enzyme expressed by HCC and is the rate-limiting enzyme that catalyzes the metabolism of Tryptophan (Trp) along the kynurenic acid (Kyn) pathway, which leads to the inhibition of T cells and is responsible for tumor cells to escape monitoring and clearance of the immune system (6). IDO1 could be up-regulated by some cytokines and immune checkpoint molecules, such as interferon-γ (IFN-γ), Toll-like receptor (TLR) 3, TLR4, immune checkpoint (including PD-1, CTLA4, CD47) to escape the immunosuppressive environment through the Janus kinase1/signal transducers and activators of transcription1 (JAK1/STAT1) pathway (7, 8). IFN-γ is one of the most essential cytokines in regulating the immune system, through up-regulating inhibitory immune checkpoints such as PD-L1 and CD47 (9). CD47 is a transmembrane protein of the immunoglobulin (Ig) superfamily and is overexpressed in several cancers, which could directly bind with SIRPα to deliver the “don’t eat me” signal that exerts anti-phagocytosis function (10, 11). Studies have shown that IFN-γ-induced CD47 upregulation is a common phenomenon in human cancers, and the JAK1-STAT1 axis is the main pathway (12).

Abrine is the dominant alkaloid extracted from *Abrus cantoniensis* and *Abrus precatorius* Linn with significant functions of protecting the liver and is a specific IDO inhibitor (13). In this study, we evaluated the effect of Abrine on anti-HCC and immune response and determined that Abrine as an IDO1 inhibitor could inhibit IFN-γ, PBMCs, or IDO1-induced IDO1-JAK1-STAT1 signaling pathway, enhanced the phagocytosis of macrophages through inhibiting CD47 expression, and decreased the PD-L1 expression in HCC cells. In the HCC xenograft mice model, Abrine suppressed the tumor growth, and promote the anti-HCC effect of anti-PD-1 antibody through increasing the infiltration of CD8^+^ T cells, decreasing Treg cells, and inhibiting PD-L1, and CD47 expression. In addition, we found that Abrine could significantly decrease the m^6^A RNA methylation in IFN-γ-induced HepG2 cells, which meant that m^6^A RNA methylation may play a role in Abrine suppression HCC.

## 2 Materials and methods

### 2.1 Cell culture

HepG2 (Human liver cancer) cells were from American Type Culture Collection (ATCC, USA), and were cultured in DMEM (Gibco, USA) medium complemented with 10% fetal bovine serum (FBS) (Gibco, USA) and 1% penicillin/streptomycin (Gibco, USA). Human peripheral blood mononuclear cells (PBMCs) were isolated from whole blood through Human Peripheral Blood Mononuclear Cell Isolation Kit (Solarbio, China) and cultured in RPMI-1640 with 15% FBS and 1% penicillin/streptomycin. The mentioned cells were maintained in 37°C incubator filled with 5% CO2. PBMC was cultured in 10% human serum RPMI-1640. The PBMC was extracted from healthy volunteers, which was approved by the Ethics Committee of Guangxi University of Chinese Medicine.

### 2.2 Cell viability assay

The cell viability was detected by MTT assay. HepG2 cells were seeded into 96-well plates and cultured over 16 hours. Then, Abrine (Chengdu Pufei De Biotech Co., Ltd, China, the purity is over 98%) at different concentrations from 5 to 40 μM was added to the HepG2 cells for 24 hours. After which 100 μL 1mg/mL MTT (Solarbio, China) reagent was added and incubated for another 4 hours at 37°C. After incubation, cells were treated with DMSO for 15 minutes at room temperature. Absorbance was measured at OD = 490 nm by a MicroplateReader (BioTeK, USA).

### 2.3 Enzyme-linked immunosorbent assay

HepG2 Cells were plated into 6 cm dishes overnight. Consequently, cells were pretreated with Abrine (0, 10, 20, and 40 µM) for 1 h, following co-incubation with IFN-γ (20 ng/mL), PBMC, or IDO1(30 ng/mL) for 24 h. The medium was collected for the determination of IFN-γ or Kyn by the ELISA Kits (Fankew, China) following the manufacturer’s instructions.

### 2.4 m^6^A modification of mRNA analysis

Buffer, S1 nuclease (Takara, Japan), phosphodiesterase (Sigma-Aldrich, USA), and alkaline phosphatase (Takara, Japan) were added into 1 µg of total RNA to completely digest RNA into nucleic acid at 37°C, then extracted with chloroform (Sinopharm Chemical Reagent co., Ltd. China) and took the upper layer water sample for subsequent LC-ESI-MS/MS analysis. The liquid phase conditions are as follows: Chromatographic column: Waters ACQUITY UPLC HSS T3 C18 column (1.8 µm, 100 mm × 2.1 mm i.d.); gradient elution program: 0 min A/B is 95:5 (V/V), 1.0 min A/B is 95:5 (V/V), 9.0 min A/B is 5:95 (V/V), 11.0 min A/B is 95:5 (V/V), 11.1 min A/B is 95:5 (V/V), 14.0 min A/B is 95:5 (V/V) (phase A is ultrapure water (2mM ammonium bicarbonate), phase B is methanol (2mM ammonium bicarbonate); flow rate was at 0.3 mL/min; column temperature is 40°C; the injection volume is 10 µL. Then the MS/MS analysis conditions are as follows: Electrospray Ionization (ESI) temperature is 550°C, mass spectrometer voltage is 5500v under the positive ion mode, and Curtain Gas (CUR) is 35 psi. In Q-Trap 6500+ (SCIEX, USA), each ion pair is scanned according to optimized Declustering Potential (DP) and Collision Energy (CE). Finally, build an MWDB (Metware Database) database based on the standard, and perform qualitative analysis on the data detected by mass spectrometry.

### 2.5 Western blot

The process of western blot was described before (14). Antibodies for GAPDH (#8884), IDO1 (#86630S), PD-L1 (#13684T), p-STAT1 (#9167S), STAT1 (# 14994S), JAK1 (#3332S) and the secondary antibodies including horseradish peroxidase (HRP)-conjugated goat anti-rabbit IgG (#7074) were purchased from Cell Signaling Technology (Beverly, MA, USA). CD47 (#ab284132) was obtained from Abcam (Cambridge, MA, USA).

### 2.6 Fluorescence assay

HepG2 cells were pretreated with or without 20 μM Abrine followed by IFN-γ (20 ng/mL), PBMCs or IDO1 treatment for 24 h. Then cells were washed with PBS for 2 times and fixed with 4% PFA for 20 min at room temperature. Cells were permeabilized with 0.5% Triton X-100 and subsequently blocked with 5% BSA for 30 min. After that, the cell samples were incubated with the primary antibody against IDO1, PD-L1, STAT1, or CD47 (1:100 dilution, 100 μL) at 4 °C overnight. Cells were then incubated with the Coralite 488 Goat Anti-Rabbit lgG (1:200 dilution, 100 μL) (SA00013-2, proteintech, China) or Coralite594 Goat Anti-Rabbit lgG (SA0001d-4, proteintech, China) for 2 h at room temperature. Immunofluorescence images were captured by the confocal laser scanning microscope (Leica TCS SP8, Solms, Germany) after staining with Hoechst 33342 for 5 min.

### 2.7 Flow cytometry

The HepG2 cells were collected after Abrine and IFN-γ, PBMC, or IDO1 treatment for 24 h. Resuspend cells (5×10^5^) in 100 μL of diluted primary antibody including IDO1, PD-L1, and CD47 respectively incubated for 0.5 h on ice and protect from light. Afterward, cells were washed with PBS and resuspended cells in 200-500 μL of PBS and analyzed by FACSMelody™ Cell Sorter (BD bioscience, USA).

### 2.8 Cellular thermal shift assay

The HEK293T cells were lysed with RIPA Lysis Buffer (1% PMSF and 1% cocktail). The respective cell lysates were co-incubated with vehicle control (DMSO) or Abrine (20 μM) for 0.5 h on ice and then centrifuged at 15,000 rpm for 20 minutes at 4°C. After which the supernatant was divided into 6 parts on average and heated respectively at different temperatures (44, 48, 52, 56, 60, and 64 °C) for 3 minutes followed by cooling for 30 s at room temperature, then detected by western blot assay (15).

### 2.9 Molecular Docking

The 2D structure of Abrine in sdf format was obtained from the PubChem database and transformed into a three-dimensional structure using ChemBio3D energy minimization saved as mol2 format. Next, the PDB number of IDO was got from the RCSB PDB database, download the 3D structure of IDO and use PyMol software to delete the water molecule and the original ligand. Hereafter, Using IDO protein as receptor and Abrine molecule as ligand, the active sites of molecular docking were determined according to the coordinates of the ligands in the target protein complex, and AutoDock Vina was used for molecular docking, then PyMol was used for correlation mapping.

### 2.10 Molecular interaction analysis

Biacore X100 (Cytiva, United States) was used for the measurement of the interaction of Abrine with IDO1. Using HBS-EP buffer (Cytiva, United States) as the working buffer, dilute the IDO recombinant protein (Sino Biological, China) to a final concentration of 20 μg/ml. Next, The surface of the CM5 chip was activated with a mixture of 0.2 mol/L EDC and 50 mmol/L NHS at a ratio of 1:1 injected continuously at a flow rate of 10 μl/min for 7 minutes, and then injected 20 ug/mL IDO recombinant protein to be coupled to CM5 chip by amino coupling method, after which 1 mol/L ethanolamine hydrochloride (pH 8.5) blocking solution was injected for 7 min to block the activated chip surface. What’s more, Abrine was diluted with HBS-EP buffer to 100, 50, 25, 12.5, 6.25, 3.125, and 1.5625 nmol/L, kinetic experiments were performed using the kinetic and affinity methods in the template of the Kinetic Analysis Wizard to analyze the interaction between the ligand and the receptor. The obtained data were fitted according to the analysis software, with time as the abscissa and the response value as the ordinate to calculate the binding kinetics between Abrine and IDO1.

### 2.11 Phagocytosis assay

The macrophages were labeled with Calcein-AM (5 μM) and incubated at 37°C in the dark for 20 min, then co-cultured with HepG2 cells labeled with pHrodo Red (120 ng/mL) at the ratio of 1:2, then added 20 μM Abrine and incubated at 37°C in the dark for 2 h. The phagocytosis of macrophages was detected by fluorescence microscope. HepG2 cells were co-cultured with macrophage cells at a ratio of 1:2 (HepG2: macrophages) and treated with or without Abrine. After 2 h, the nanolive label-free system was used to in real time observe the effect of Abrine on macrophage cells engulfing tumor cells. The videos were processed with image J.

### 2.12 Gene expression profiling

To further reveal the role of IFN-γ in hepatic carcinoma, the correlations between IFN-γ and CD47, IDO1, and PD-L1 were analyzed by calculating Pearson correlation coefficients through The Cancer Genome Atlas (TCGA) database, the cBioPortal website (https://www.cbioportal.org/) and UCSC Xena (https://xena.ucsc.edu/) website. The correlation between IFN-γ and CD47, IDO1, and PD-L1 was analyzed by calculating Pearson correlation coefficients in GraphPad Prism 9.

### 2.13 Tumor xenografts animal experiments

Animal experiments were approved by the Ethics Committee on Laboratory Animal Management of Guangxi University of Chinese Medicine (Approval Document No. SYXK-2019–0001). Healthy C57BL/6J mice (SPF degree, 6-8 weeks old, male, weight 18-22 g) were purchased from Vital River Laboratory (Guangdong, China, animal license #: SCXK-2022-0063). All animals were housed under specific pathogen-free (SPF) conditions at 25°C with 50% humidity and free access to food and water. After three days of adaptive feeding, Hepa1-6 cells in 0.1 mL basic DMEM were inoculated in the right hind leg of the mice at a density of 1 × 10^6^ cells/mice apart from those in the control group (16, 17). After 7 days, the tumor volume reached almost 100 mm^3^ in mice. The tumor-bearing mice were randomly divided into 4 groups (n = 6 for each group): the model group, the anti-PD-1 Ab group, the Abrine group (15 mg/kg) (18), and the combination of Abrine and anti-PD-1 Ab group. The unvaccinated mice served as a control group (n=6). On day 7, Abrine was dissolved with saline and administered into mice by intraperitoneal injection (i.p.) for 14 days, once a day. At the same time, anti-PD-1 Ab (Purity>95%, InVivoMab anti-mouse PD-1 (CD279), BioXCell, USA) was freshly prepared by PBS and intraperitoneally injected into mice (200 μg/mice), once every 3 days (19). Besides, the mice in the control and model groups were injected with an equal volume of saline. The mice’s tumor volume was measured every two days. On day 21, the blood, tumor, and organ tissues of mice were collected after the mice were anesthetized with isoflurane and sacrificed.

### 2.14 Flow cytometry of tumor tissues

The single cell suspension from tumor tissues was filtered after subsequently resuspended for counting and concentration adjustment, labeled with biomarkers CD45-PerCP-Cy™5.5 rat anti-mouse (#550994, BD Biosciences, USA), CD3-BV510 hamster anti-mouse (#740113, BD Biosciences, USA), CD4-PE-Cy™7 rat anti-mouse (#552775, BD Biosciences, USA) and CD8a-BV786 rat anti-mouse (#563332, BD Biosciences, USA) for subsequent flow cytometry detection.

### 2.15 Hematoxylin and eosin staining, single- and multiplex immunofluorescence

After the mice were anesthetized, the heart, liver, spleen, lung, kidney, brain tissue, and part of tumor tissue specimens were isolated, and fixed in 4% Paraformaldehyde Fix Solution for HE staining, single- and multiplex immunofluorescence for CD47, IDO1, CD8, PD-L1, and Foxp3. The rest of the tumor was frozen in liquid nitrogen for other studies.

### 2.16 Statistics

Student’s unpaired t-test and one-way ANOVA in GraphPad Prism were used for statistical analysis in GraphPad Prism 9 (GraphPad Software, USA). *P* < 0.05 were considered statistically significant.

## 3 Results

### 3.1 Abrine inhibits m^6^A RNA methylation and IDO1/JAK1/STAT1 signal pathway in IFN-γ-induced HepG2 cells

**Figure 1:**
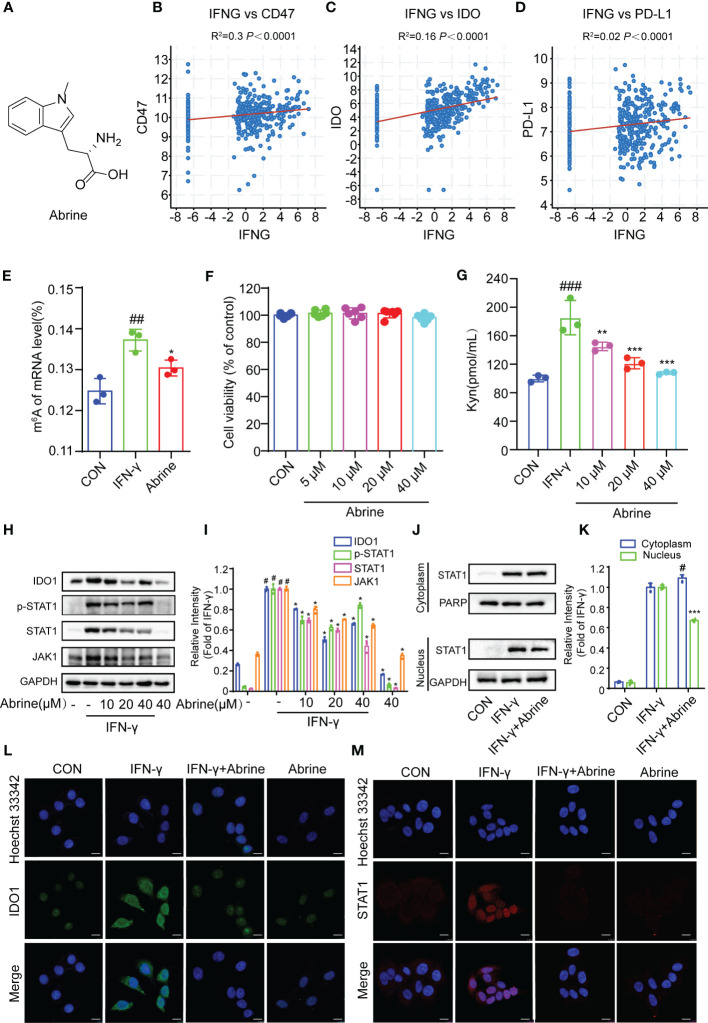
Abrine inhibits m^6^A RNA methylation and IDO1/JAK1/STAT1 signal pathway in IFN-γ-induced HepG2 cells. **(A)** The structure of Abrine; **(B–E)** The correlations between IFN-γ and CD47, IDO, and PD-L1 in HCC cells were analyzed by calculating the Pearson correlation coefficient; **(E)** The RNA m6A methylation analysis based on LC-MS/MS, ^##^*p* < 0.01 versus the control group; ^*^p < 0.05 versus the IFN-γ group; **(F)** HepG2 cells were treated with the Abrine for 24 **h** The cytotoxicity was determined by MTT assay; **(G)** The effect of Abrine on the Kyn levels in IFN-γ-induced HepG2 cells by ELISA assay, ^###^*p* < 0.001 versus the control group; ^**^p < 0.01, ^***^p < 0.001 versus the IFN-γ group; **(H, I)** Western blot analysis detects the effect of Abrine on the expression of the proteins including IDO1, p-STAT1, STAT1, and JAK1. The relative protein band intensities were counted, ^#^*p* < 0.001 versus the control group; ^*^p < 0.001 versus the IFN-γ group; **(J, K)** The localization of STAT1 in the cytoplasm and nucleus of HepG2 cells was detected by western blotting, ^#^*p* < 0.05 versus the control group; ^***^p < 0.001 versus the IFN-γ group; **(L, M)** The immunofluorescence detected the effect of Abrine on the expression of IDO1 and STAT1 translocation in IFN-γ-induced HepG2 cells (Scale bar = 20 μm).

Abrine is a natural product extracted from Traditional Chinese Medicine (**Figure 1A**). To explore the relationship between IFN-γ and the expression of immune checkpoints, the correlation between IFN-γ and CD47, IDO1, and PD-L1 was detected from the TCGA database, and it was found that IFN-γ had positive correlation responses with all of them in HCC (**Figures 1B–D**). The m^6^A RNA methylation modification plays an important role in the occurrence and progression of cancers, in this study, we found that IFN-γ treatment increased the m^6^A RNA methylation in HepG2 cells, while Abrine inhibits IFN-γ-induced RNA m^6^A methylation (**Figure 1E**). At the same time, Abrine at 5, 10, 20, and 40 μM has no cytotoxic in HepG2 cells. (**Figure 1F**). In addition, Abrine inhibited Kynurenine (Kyn) level, which was produced by the metabolism of tryptophan through the activation of the key metabolic enzyme IDO1 (**Figure 1G**). Abrine decreased the protein expression of IDO1, JAK1, p-STAT1, and STAT1 (**Figures 1H, I, L**) in IFN-γ-induced HepG2 cells. Moreover, Abrine inhibited the translocation of STAT1 from the cytoplasm into nuclear (**Figures 1J, K, M**). These data indicated that Abrine has an epigenetic regulatory role and inhibits IDO1/JAK1/STAT1 signaling pathway in IFN-γ-induced HepG2 cells.

### 3.2 Abrine inhibits IDO-1/JAK1/STAT1 signal pathway in PBMC-induced HepG2 cells

**Figure 2:**
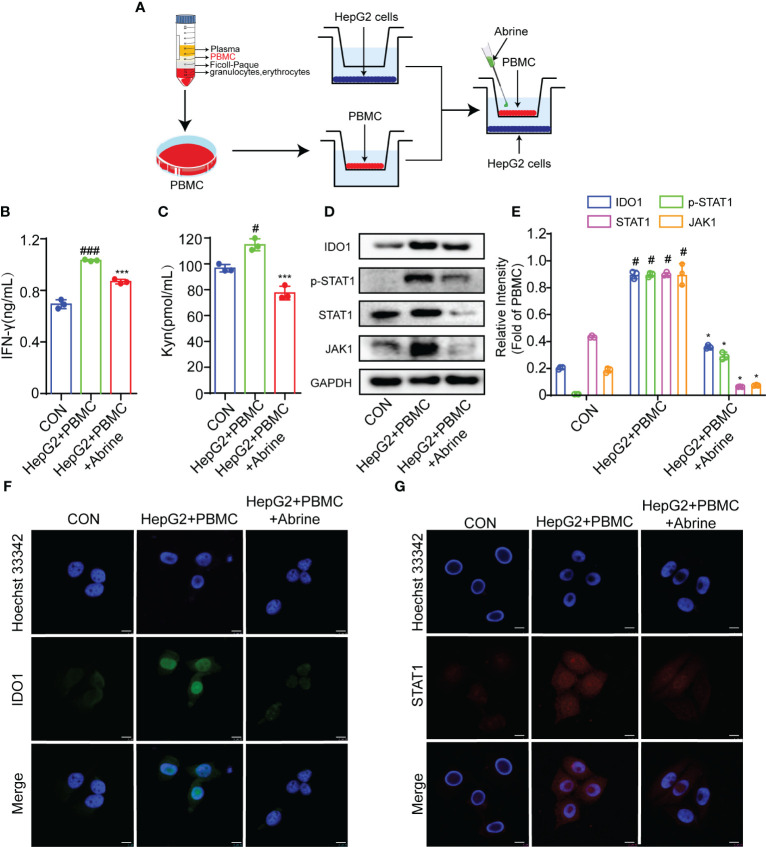
Abrine inhibits IDO-1/JAK1/STAT1 signal pathway in PBMC-induced HepG2 cells. **(A)** The schedule of PBMC co-culture with HepG2 cells; **(B, C)** The effect of Abrine on the level of IFN-γ and Kyn in PBMC-induced HepG2 cells; ^#^*p* < 0.05, ^###^*p* < 0.001 versus the control group; ^***^p < 0.001 versus the HepG2+PBMC group; **(D, E)** The effect of Abrine on the proteins expressions in PBMC-induced HepG2 cells as indicated; ^#^*p* < 0.001 versus the control group; ^*^p < 0.001 versus the HepG2+PBMC group; **(F, G)** Immunofluorescence detected the effect of Abrine on the expression of IDO1 and STAT1 in PBMC-induced HepG2 cells (Scale bar = 20 μm).

The co-culture model of immune cells and tumor cells is the most widely used model of tumor immunity research *in vitro.* In this study, PBMCs were co-cultured with HepG2 cells to imitate the interaction between immune cells and tumor cells, and to better explore the effectiveness and internal mechanism of tumor immunity research strategies (**Figure 2A**). As shown in **Figures 2B, C**, PBMCs treatment increased the IFN-γ and Kyn level, which was suppressed by Abrine. What’s more, the expression of IDO1, JAK1, p-STAT1, and STAT1 was determined by western blot. The results showed that the expressions of IDO-1, JAK1, p-STAT1, and STAT1 proteins were increased in PBMCs co-cultured HepG2 cells, while Abrine inhibited the protein expression (**Figures 2D–F**). Besides, Abrine suppressed the STAT1 translocation from the cytoplasm into the nuclear (**Figure 2G**), these data indicated that PBMC increased the IFN-γ and Kyn level in HepG2 cells, therefore increasing the IDO1/JAK1/STAT1 signaling pathway proteins expression, which was suppressed by Abrine.

### 3.3 Abrine targets on IDO1 to inhibit IDO1/JAK1/STAT1 signaling pathway

**Figure 3:**
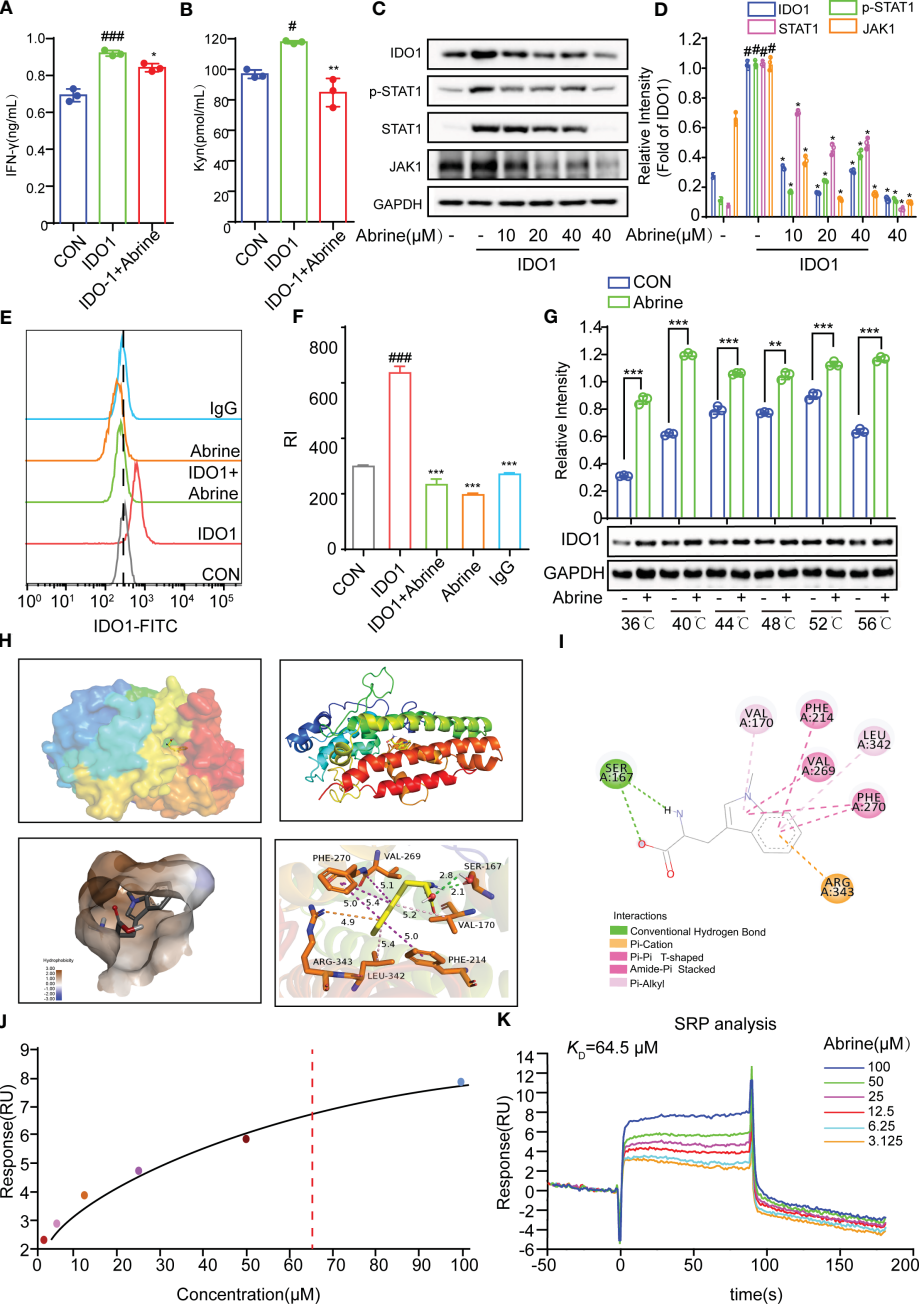
Abrine targets on IDO1 to inhibit IDO1/JAK1/STAT1 signaling pathway. **(A, B)** The effect of Abrine on the level of IFN-γ and Kyn in IDO1-induced HepG2 cells, ^#^*p* < 0.05, ^###^*p* < 0.001 versus the control, ^*^p < 0.05, ^**^p < 0.01versus the IDO1 group; **(C, D)** The effect of Abrine on the expression of the proteins in IDO1-induced HepG2 cells as indicated, ^#^*p* < 0.001 versus the control, ^*^p < 0.001 versus the IDO1 group; **(E, F)** Flow cytometry detected the level of IDO-1 in IDO-1-induced HepG2 cells, ^###^*p* < 0.001 versus the control, ^***^p < 0.001 versus the IDO1 group; **(G)** CETSA detected the interaction of Abrine with IDO1; **(H, I)** Molecular docking results of Abrine with IDO1, ^**^p < 0.01, ^***^p < 0.001; **(J, K)** Biacore X100 detected the kinetics/Affinity of Abrine with IDO-1.

IDO1, the first rate-limiting enzyme of tryptophan catabolism, is continuously highly expressed in a variety of solid tumor tissues and is closely related to poor prognosis. The inhibition of IDO1 can promote the efficacy of immunization and chemotherapy (20). Therefore, IDO1 inhibitors have a prospect for development as potential drugs for tumor immunotherapy. Abrine has been reported to be a specific IDO1 inhibitor. In the present study, Abrine suppressed the increased IFN-γ and Kyn levels in IDO1 recombinant protein-treated HepG2 cells (**Figures 3A, B**), and increased the expression of IDO1, JAK1, p-STAT1, and STAT1 in HepG2 cells, while all of which were decreased by Abrine (**Figures 3C, D**). What’s more, flow cytometry results further indicated that increased IDO1 expression was inhibited by Abrine in IDO1-induced HepG2 cells (**Figures 3E, F**). Afterward, the molecular docking assay was used to examine the interaction between Abrine and IDO1. The results showed that Abrine interacts with IDO1 at the sites of SER:167, VAL:170, PHE:214, LEU:342, VAL:269, PHE:270, and ARG:343 (**Figures 3H, I**). Biacore X100 SPR assay was used to detect the Kinetics/Affinity of Abrine and IDO1, results showed that the *K*_D_ value was 64.5 μM, which indicated that Abrine has a strong interaction ability with IDO1 (**Figures 3J, K**). CETSA assay further confirmed that IDO1 protein was more stable under the action of Abrine at a series of temperatures (**Figure 3G**). These data indicated that Abrine interacts with IDO1 and as an inhibitor of IDO1 to inhibit IDO1/JAK1/STAT1 signaling pathway in IDO-1-induced HepG2 cells.

### 3.4 Abrine inhibits CD47 and promotes the phagocytosis of macrophages

**Figure 4:**
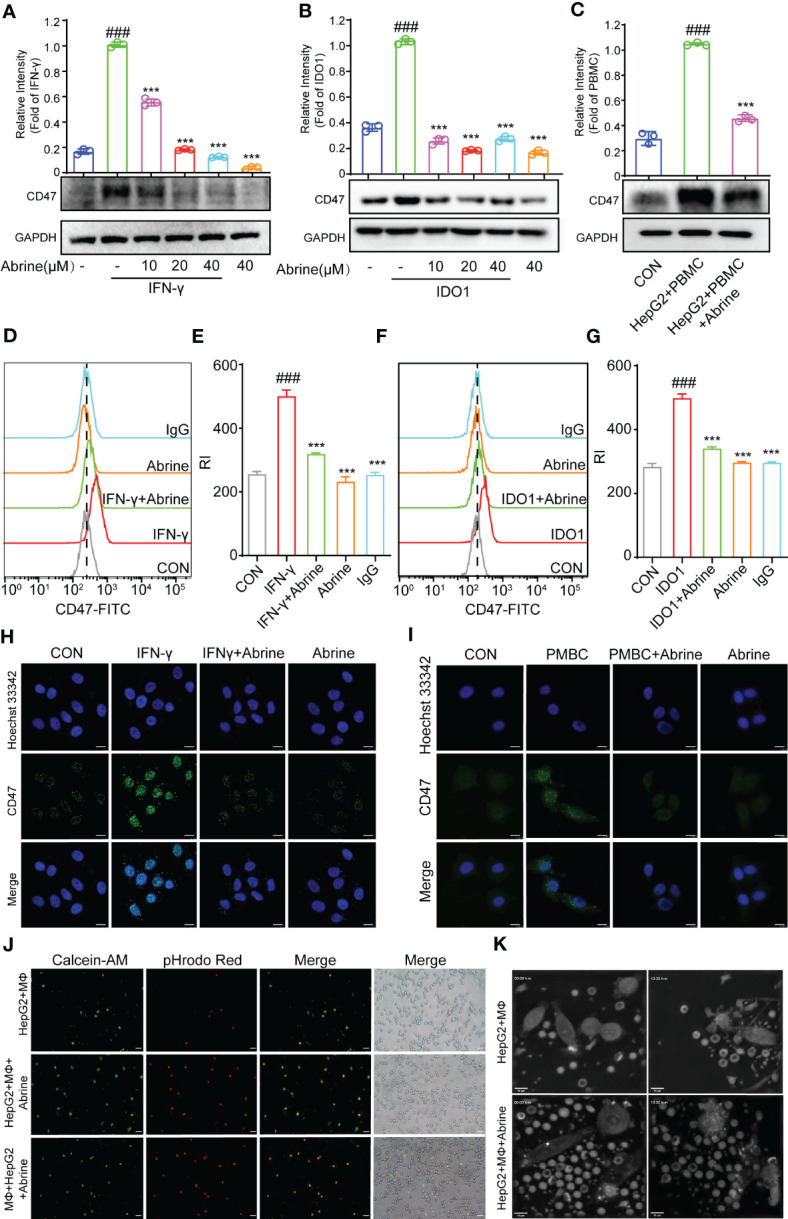
Abrine inhibits CD47 and promotes the phagocytosis of macrophages. **(A–C)** Western blot detected the expression of CD47 in IFN-γ, IDO1, and PBMC-induced HepG2 cells; **(D–G)** Flow cytometry detected the level of CD47 in IFN-γ and IDO1-induced HepG2 cells; **(H, I)** Immunofluorescence detected the expression of CD47 in IFN-γ and PBMC-induced HepG2 cells (Scale bar = 20 μm); **(J)** The phagocytosis effect of Abrine on macrophages engulf the HepG2 cells (Scale bar = 20 μm). **(K)** Images from Video 1 and 2, HepG2 cells were co-cultured with macrophages at a ratio of 1:2, and the phagocytosis effect of Abrine on macrophages engulf the HepG2 cells after co-culture 2 h was detected by the nanolive label-free system (Scale bar = 10 μm). ^###^*p* < 0.001 versus the control, ^***^p < 0.005 versus the model group.

CD47 is an important anti-phagocytosis signal, which can prevent the phagocytosis of tumor cells by macrophages *via* binding to ligand signal regulatory protein α (SIRPα) on macrophages (21). We found a positive correlation between interferon-γ (IFN-γ) and CD47 in HCC cells (**Figure 1B**), IFN-γ, PBMCs, and IDO1 could increase the expression of CD47 in HepG2 cells, while Abrine decreased the expression of CD47 (**Figures 4A–C**). The CD47 expression was further detected by flow cytometry and immunofluorescence, results indicated that Abrine could decrease the expression of CD47 in IFN-γ, PBMCs, and IDO1-induced cells (**Figures 4D–I**). Then, Calcein-AM-labeled macrophages derived from PBMC, and pHrodo Red-labeled HepG2 cells were co-cultured to detect the phagocytosis of macrophages to tumor cells. Results showed that Abrine treatment in either HepG2 or macrophages both increased the phagocytosis effect of macrophages on HepG2 cells and could recruit more macrophages to the cancer cells (**Figures 4J, K** and Video1, 2). Collectively, these data suggested that Abrine could promote the phagocytosis of tumor cells by macrophages and prevent the immune escape of tumor cells by inhibiting the expression of CD47.

### 3.5 Abrine inhibits PD-L1 in IDO1 overexpression HepG2 cells

**Figure 5:**
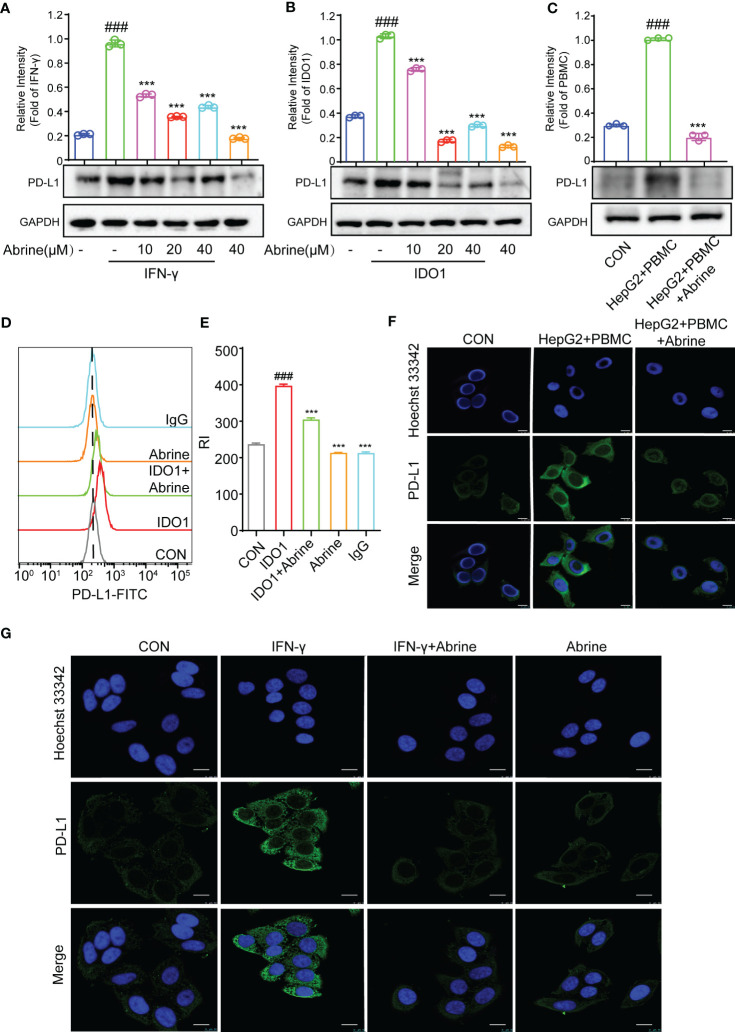
Abrine inhibits PD-L1 in IDO1 overexpression HepG2 cells. **(A–C)** Western blot detected the expression of PD-L1 in IFN-γ, IDO1, and PBMC-induced HepG2 cells; **(D, E)** Flow cytometry detected the level of PD-L1 in IDO1-induced HepG2 cells; **(F, G)** Immunofluorescence detected the expression of PD-L1 in PBMC and IFN-γ-induced HepG2 cells (Scale bar = 20 μm). ^###^*p* < 0.001 versus the control, ^***^p < 0.005 versus the model group.

PD-L1/PD-1 axis is an important immune checkpoint, which can promote the tumor cell escape from immune monitoring, and the PD-L1/PD-1 inhibitors as ICIs are widely used in clinical for the treatment of varieties of cancers (22). In this study, we found a positive correlation between interferon-γ (IFN-γ) and PD-L1 in HCC cells (**Figure 1B**), and this study found that Abrine could decrease the PD-L1 expression in IFN-γ, PBMCs, and IDO1-induced IDO1 overexpression HepG2 cells (**Figures 5A–C**). The flow cytometry results indicated that IDO1 increased the expression of PD-L1, Abrine could decrease the expression of PD-L1 (**Figures 5D, E**). The immunofluorescence results showed that PBMC or IFN-γ increased the expression of PD-L1, and Abrine suppressed its expression (**Figures 5F, G**). These data indicated that IDO1 overexpression may lead to the increased expression of PD-L1, which could be inhibited by the IDO1 inhibitor Abrine.

### 3.6 Abrine and anti-PD-1 antibody treatment has a synergistic effect on Hepa1-6 xenograft mice model

**Figure 6:**
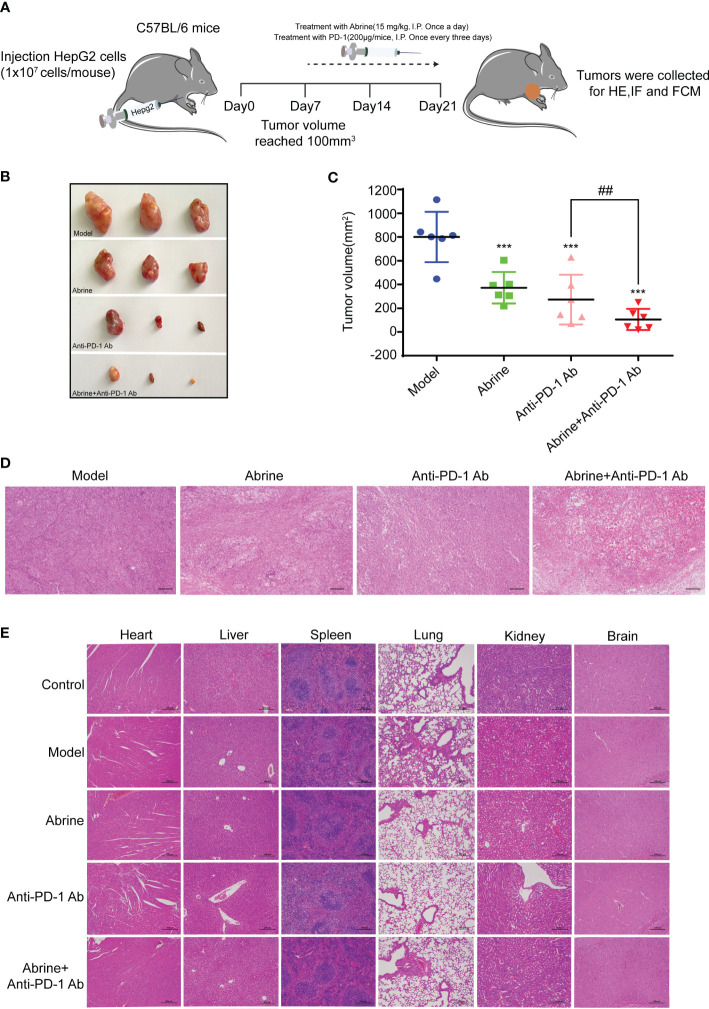
Abrine and anti-PD-1 antibody treatment has a synergistic effect on Hepa1-6 xenograft mice model. **(A–C)** The effect of Abrine, anti-PD-1 antibody, and Abrine co-culture with anti-PD-1 antibody on tumor size, tumor volume, and pathological changes; **(D)** The HE staining of tumor tissues (Scale bar = 20 μm). **(E)** The HE staining of heart, liver, spleen, lung, kidney, and brain (Scale bar = 20 μm). ^***^p < 0.005 versus the model group, ^##^*p* < 0.01 versus the anti-PD-1 Ab group.

**Figure 7:**
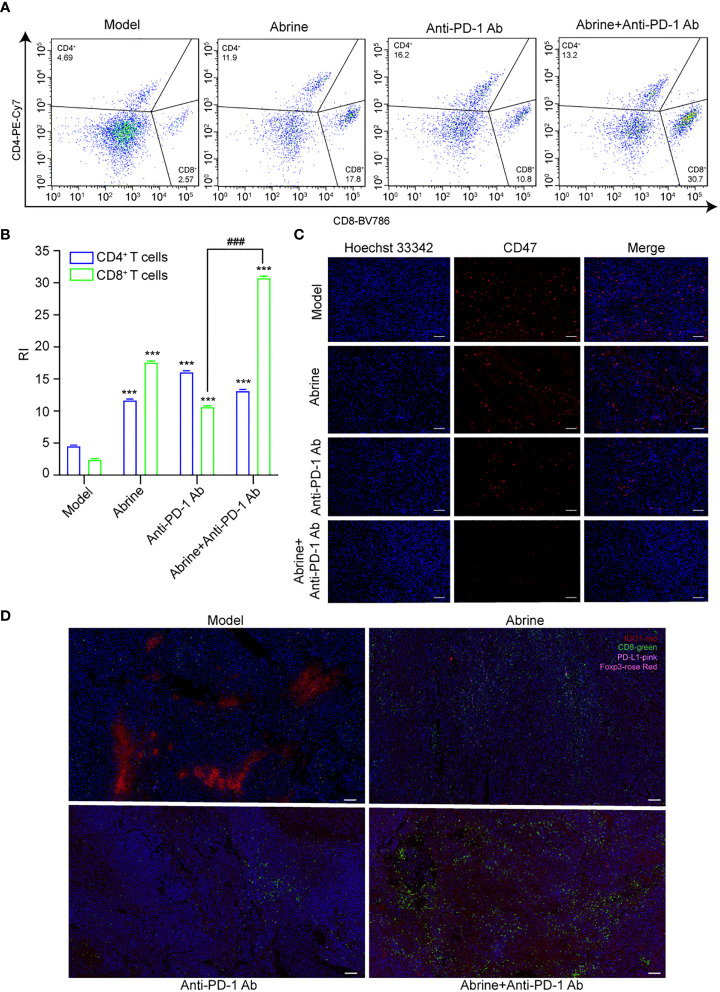
Abrine and anti-PD-1 antibody treatment has a synergistic effect on Hepa1-6 xenograft mice model. **(A, B)** Flow cytometry detected the effect of Abrine, amti-PD-1 antibody, and Abrine co-culture with anti-PD-1 antibody on CD4^+^ T cells and CD8^+^ T cells; **(C)** The IHC staining of CD47 expression (Scale bar = 20 μm); **(D)** The mIHC staining detect the expression of CD8+ T cells, PD-L1, IDO1, Foxp3 expression in tumor tissues (Scale bar = 10 μm). ^***^p < 0.005 versus the model group, ^###^*p* < 0.001 versus anti-PD-1 Ab group.

Although anti-PD-1 immunotherapy has great progress in tumor treatment, there are still problems such as low response rate and adverse reactions in the treatment of many solid tumors. The high expression of IDO1 is also the main cause of resistance to PD-1/PD-L1 inhibitors. Based on this, we combined Abrine with anti-PD-1 antibody to treat the Hepa1-6 xenograft mice model. Results showed that Abrine, anti-PD-1 antibody, and the combination treatment groups could suppress the tumor growth and tumor volume, and Abrine co-treated with anti-PD-1 antibody has a synergistic effect on inhibiting the tumor growth than Abrine or anti-PD-1 antibody-treated groups (**Figures 6A–D**). And HE staining of the heart, liver, spleen, lung, kidney, and brain showed the safety of Abrine (**Figure 6E**). Flow cytometry detected the CD3^+^CD4^+^ T cells and CD3^+^CD8^+^ T cells, results indicated that Abrine co-treated with anti-PD-1 antibody increased the CD3^+^CD8^+^ T cells obviously than Abrine or anti-PD-1 antibody-treated groups, which means that the infiltration of CD8^+^T cells was increased in tumor cells and promotes immune responses (**Figures 7A, B**). As shown in **Figure 7C**, CD47 expression increased in model mice, while Abrine, anti-PD-1 antibody, and the combination treatment groups could suppress the expression of CD47, and the combination treatment groups has a better suppression effect than Abrine or anti-PD-1 antibody-treated groups. mIHC results showed that Abrine co-treated with anti-PD-1 antibody increased CD8^+^ cytotoxic T cells infiltration in tumor cells, decreased Foxp3^+^ Treg cells, and inhibited IDO1 and PD-L1 expression (**Figure 7D**). These data indicated that Abrine has a synergistic effect with the anti-PD-1 antibody on the treatment of HCC through regulating immune responses.

## 4 Discussion

Due to the complex pathogenesis, high molecular heterogeneity and immune tolerance microenvironment, the systemic treatment of advanced liver cancer has always been a difficult research point (23–25). In recent years, immunotherapy especially immune checkpoint inhibitors (ICIs) have brought a new opportunity to improve the survival rate of patients with advanced liver cancer (26). However, the related therapeutic drugs and mechanisms still need more research. In this study, we found that Abrine as an IDO1 inhibitor has an inhibition effect on immune escape, and its combination therapy with immune checkpoint inhibitor anti-PD-1 antibody exerted a better anti-tumor effect.

IDO1 expression is present not only in tumor cells but also in endothelial cells, fibroblasts, and immune cells that infiltrate the TME (27). The main function of IDO1 is to decompose Trp into Kyn and its downstream metabolites, which are responsible for tumor immune escape by regulating T cell-associated immune responses and promoting the activation of immunosuppressive cells (28). Studies have shown that most tumor cells are positive for IDO1, and the strong expression of IDO1 in tumor tissue has also been identified as an independent negative prognostic factor for many cancers (29–32). IDO1 expression of tumor cells correlates with tumor-infiltrating Foxp3^+^ Tregs and other immunosuppressive molecules such as PD-1 and its ligand PD-L1 (33). IFN-γ is widely considered to be the major inducer of IDO1. As HepG2 cells hardly express IDO1, co-cultured with IFN-γ or PBMCs could be upregulated (34). Our data showed that Abrine significantly reduced the expression of IDO1 and Kyn level in IFN-γ, PBMCs, or exogenous IDO1-induced HepG2 cells.

IFN-γ is one of the most important cytokines in inflammatory and immune responses, mainly produced by natural killer (NK) cells in the innate immune system and T cells in the adaptive immune system, which plays an important role in immunopathology and immune response (35). The JAKs/STAT1 pathway is critical for IFN-γ to generate signal transduction. Binding of IFN-γ to its receptor IFNGR activates JAKs, which subsequently lead to phosphorylation, activation, and dimerization of the transcription factor STAT1. The newly formed STAT1 homodimers subsequently translocate to the nucleus where they initiate the transcription of some IFN-γ-stimulated genes (ISGs) (36, 37). Our data showed that Abrine could reduce the level of IFN-γ elevated by PBMCs or IDO1, significantly decrease the expression of JAK1 and the phosphorylation of STAT1, besides, prevent IFN-γ or PBMCs-induced nuclear translocation of STAT1.

IFN-γ regulates immune escape correlated with the overexpression of immune checkpoint receptors including PD-L1 and IDO1, which eliminates T cell activity in tumor tissues (38, 39). The combination of highly expressed PD-L1 on tumor cells and the receptor PD-1 on T cells transmit negative regulatory signals, induce T cell apoptosis, or lead to immune incompetence, therefore, tumor cells can escape from the immune monitoring and killing (40). At the same time, the activation of the PD-1/PD-L1 axis can also change the differentiation of T cells, impair the differentiation of effector T cells (Teff), memory T cells (Tm), regulatory T lymphocytes (Treg) and exhausted T cells (Tex), thereby significantly inhibiting T cell immune effects (41, 42). In addition, Ye et al. found that IFN-γ-induced increased CD47 expression through the JAK1-STAT1 axis might be a common phenomenon in cancer, which would increase the affinity between CD47 and SIRPα, amplify the “don’t eat me” signal, reduce the phagocytosis ability of macrophages, and mediate immune escape (43). TCGA database statistic results showed that IFN-γ was positively correlated with both CD47 and PD-L1 in HCC. Our further experiments found that IFN-γ or PBMCs up-regulated the levels of PD-L1 and CD47 in HepG2 cells obviously, which could be inhibited by Abrine. Moreover, the Abrine treatment also promoted the phagocytosis of HepG2 cells by macrophages, which might be related to the inhibitory effect of Abrine on CD47. However, the effect of Abrine on CD47-SIRPα signal needs further study.

PD-1/PD-L1 monoclonal antibodies have made breakthroughs in the treatment of many cancers in clinical, but there are still problems such as a high incidence of adverse reactions and a large range of treatment tolerance (44, 45). Previous studies found that the high expression of IDO1 is the main cause of resistance to PD-1/PD-L1 inhibitors (46). Therefore, IDO1 inhibitors not only exert anti-tumor activity but also may enhance the therapeutic effect of PD-1/PD-L1 inhibitors when combined with PD-1/PD-L1 inhibitors. In this study, Abrine and anti-PD-1 antibody were used to treat Hepa1-6 xenograft mice, results showed that both inhibited the expression of CD47 and PD-L1 in tumor tissues of mice, increased the levels of CD4^+^ and CD8^+^ T cells, decreased the level of Foxp3^+^ Treg cells. The combination of Abrine and anti-PD-1 antibody obtained a better tumor inhibitory effect than the two used alone, indicating that there is a synergistic effect of Abrine with anti-PD-1 antibody on the treatment of HCC.

m6A modification is an RNA-associated epigenetic regulation similar to DNA and histone modifications. Among which, m6A methylation is the most abundant epitranscriptomic modification in eukaryotic mRNA, participates in the complex and fine biological regulation of important functional genes in many cellular activities, and may promote carcinogenesis by up-regulating or down-regulating important components of cell signal transduction in the occurrence and development of cancer (47–50). Studies have shown that JAKs-STAT1 signaling pathway may be regulated by m6A at the transcriptional level, resulting in aberrant signaling in cancer progression. Suppressors of cytokine signaling (SOCS) are negative regulators of the JAKs-STAT1 signaling pathway, inhibiting the activation of this pathway under normal physiological conditions (51, 52). In HCC, SOCS is recognized and degraded as a target of m6A writer methyltransferase-like 3 (METTL3)-mediated m6A modification, thereby abrogating its inhibitory effect on the JAKs-STAT1 pathway (53, 54). These findings show that m6A methylation modification has a regulatory effect on the JAKs-STAT1 pathway in HCC progression. In the study, we found that IFN-γ can induce the increase of m6A modification in HepG2 cells, and this increased m6A methylation level was significantly inhibited by Abrine, indicating that abrine has a role in regulating abnormal m6A modification in tumor cells, therefore affecting the JAK1/STAT1 signal pathway.

Collectively, we found that Abrine has an anti-tumor immune escape and promote immune response effect by inhibiting IDO1. Abrine targets IDO1 to down-regulate the level of IFN-γ and the accumulation of metabolite Kyn, inhibiting the expression of PD-L1 and CD47 through the JAK1-STAT1 signaling pathway. In addition, Abrine synergizes with immune checkpoint inhibitor anti-PD-1 antibody to enhance tumor suppression, increases the infiltration of CD8^+^ T cells in the tumor cells, decreases the expression of CD47 and PD-L1 in tumor tissues, and down-regulate the Foxp3^+^ Treg cells to exert anti-tumor immune escape.

## Data availability statement

The original contributions presented in the study are included in the article/**Supplementary Material**. Further inquiries can be directed to the corresponding authors.

## Ethics statement

The studies involving human participants were reviewed and approved by the Ethics Committee of Guangxi University of Chinese Medicine. The patients/participants provided their written informed consent to participate in this study. The animal study was reviewed and approved by Ethics Committee on Laboratory Animal Management of Guangxi University of Chinese Medicine.

## Author contributions

HG and JX designed the research. XL, SH, and JH conducted experiments in vitro. XL and RY conducted experiments in vivo. RY and XL wrote the manuscript. C. Yao and SY revised the manuscript. All authors reviewed the manuscript.
